# Host plant‐related genomic differentiation in the European cherry fruit fly, *Rhagoletis cerasi*

**DOI:** 10.1111/mec.15239

**Published:** 2019-10-13

**Authors:** Vid Bakovic, Hannes Schuler, Martin Schebeck, Jeffrey L. Feder, Christian Stauffer, Gregory J. Ragland

**Affiliations:** ^1^ Department of Forest and Soil Sciences BOKU University of Natural Resources and Life Sciences Vienna Vienna Austria; ^2^ Department of Biology IFM University of Linköping Linköping Sweden; ^3^ Faculty of Science and Technology Free University of Bozen‐Bolzano Bolzano Italy; ^4^ Department of Biological Sciences University of Notre Dame Notre Dame IN USA; ^5^ Department of Integrative Biology University of Colorado‐Denver Denver CO USA

**Keywords:** ecological speciation, host plant races, RADSeq,Tephritidae

## Abstract

Elucidating the mechanisms and conditions facilitating the formation of biodiversity are central topics in evolutionary biology. A growing number of studies imply that divergent ecological selection may often play a critical role in speciation by counteracting the homogenising effects of gene flow. Several examples involve phytophagous insects, where divergent selection pressures associated with host plant shifts may generate reproductive isolation, promoting speciation. Here, we use ddRADseq to assess the population structure and to test for host‐related genomic differentiation in the European cherry fruit fly, *Rhagoletis cerasi* (L., 1758) (Diptera: Tephritidae). This tephritid is distributed throughout Europe and western Asia, and has adapted to two different genera of host plants, *Prunus* spp. (cherries) and *Lonicera* spp. (honeysuckle). Our data imply that geographic distance and geomorphic barriers serve as the primary factors shaping genetic population structure across the species range. Locally, however, flies genetically cluster according to host plant, with consistent allele frequency differences displayed by a subset of loci between *Prunus* and *Lonicera* flies across four sites surveyed in Germany and Norway. These 17 loci display significantly higher F_ST_ values between host plants than others. They also showed high levels of linkage disequilibrium within and between *Prunus* and *Lonicera* flies, supporting host‐related selection and reduced gene flow. Our findings support the existence of sympatric host races in *R. cerasi* embedded within broader patterns of geographic variation in the fly, similar to the related apple maggot, *Rhagoletis pomonella*, in North America.

## 1 INTRODUCTION

The mechanisms and conditions facilitating species formation and the maintenance of species boundaries are central to our understanding the evolutionary process, wherein natural or sexual selection can often drive phenotypic differentiation and adaptive radiation. The general role of natural selection per se is largely undisputed, but the population genetic details, especially the role of gene flow, continues to be hotly debated (Berlocher & Feder, 2002; Bird, Fernandez‐Silva, Skillings, & Toonen, 2012; Bolnick & Fitzpatrick, 2007; Elmer, 2019; Mallet, Meyer, Nosil, & Feder, 2009). Indeed, theoretical studies of ecological speciation have recently focused on quantifying the combined effects of population genetic processes on genetic divergence, particularly the interplay of gene flow and divergent selection (Campbell, Poelstra, & Yoder, 2018; Nosil, 2012). Much of our knowledge of ecological speciation is based on a growing number of model and non‐model organisms sampled sparsely across the tree of life including bacteria (Luo et al., 2011), fungi (Giraud, Refrégier, Le Gac, de Vienne, & Hood, 2008), plants (Macnair & Christie, 1983) and various insects (Egan et al., 2015; Nosil et al., 2018; Nouhaud et al., 2018). Nevertheless, the frequency at which ecological speciation occurs remains an open question (Feder, Egan, & Nosil, 2012; Fitzpatrick, Fordyce, & Gavrilets, 2009).

One approach to infer ecological speciation is to conduct genome‐wide surveys of co‐occurring (sympatric) populations across environments with contrasting selection pressures (Campbell et al., 2018; Vertacnik & Linnen, 2017). Additional studies can then be conducted to demonstrate that genomic regions exhibiting enhanced divergence also associate with phenotypes that are the targets of selection. Combined, these pieces of evidence allow strong inferences of differential ecological adaptation contributing to reproductive isolation (RI). Some well described examples come from phytophagous insects, where host‐associated adaptation is thought to generate post‐zygotic RI via natural selection against hybrids having intermediate phenotypes. Two examples that illustrate this scenario are the walking stick, *Timema cristinae,* and the apple maggot, *Rhagoletis pomonella* (Gloss, Groen, & Whiteman, 2016). *Timema cristinae* has two morphologically different ecotypes residing on two different host plants, *Adenostoma fasciculatum* and *Ceanothus spinosus* (Nosil, Crespi, & Sandoval, 2002; Nosil et al., 2018; Soria‐Carrasco et al., 2014). Here, bird predation drives divergent selection on morphology, selecting for *T. cristinae* individuals that are more cryptic on the host plant they originate from, and against individuals that are not cryptic on either, i.e., hybrids (Nosil et al., 2002; Sandoval, 1994; Sandoval & Crespi, 2008). Recently, however, a third melanistic ecotype that is cryptic on branches of both host plants was found to foster gene flow between *T. cristinae* ecotypes constraining divergence (Comeault et al., 2015). Nevertheless, individuals on the two host plants are genetically divergent despite evidence for gene flow (Comeault et al., 2015; Nosil & Crespi, 2007), and loci demonstrating host differences are also associated with the crypsis phenotype (Comeault et al., 2014). In *R. pomonella*, selection favours divergent seasonal timing phenotypes that synchronises flies with the availability of fruits on different host plants with varying phenologies (Bush, 1969; Dambroski & Feder, 2007). Selection favours early versus late phenology between host‐associated populations that continue to exchange genes (Feder & Filchak, 1999; Feder, Hunt, & Bush, 1993) and results in genetic divergence that is also associated with phenology phenotypes (Egan et al., 2015; Ragland et al., 2017).

When information about specific phenotypes under selection is lacking, genome surveys may still provide support for ecological divergence if the following can be established: (a) contemporary gene flow between populations in different environments; and (b) intrinsic forms of RI, such as Bateson‐Dobzhansky‐Muller incompatibilities, are largely lacking between populations. Direct evidence for migration and gene flow between habitat‐ or host‐associated populations can come from mark‐release‐recapture studies (e.g., Feder et al., 1994), an informative but difficult and time‐consuming approach. Alternatively, ongoing gene flow is often deduced indirectly from patterns of habitat‐related genetic differentiation (e.g., Soria‐Carrasco et al., 2014). In such cases, theory predicts that under the ecological speciation hypothesis, regions of the genome subject to divergent environmental selection should show significant differentiation between populations, while those that are not should be homogenised by gene flow (Nosil, 2012). To adequately establish such a pattern requires genetically surveying not just one but multiple pairs of sympatric populations inhabiting alternative environments arrayed across the landscape (e.g., Soria‐Carrasco et al., 2014). Further, with a sufficiently high rate of gene flow, local populations inhabiting alternate environments should be genetically more similar to one another than to populations associated with the same environments at geographically distant sites (e.g., Doellman et al., 2018).

The above evidence addresses gene flow, but inferring adaptive divergence between sympatric population pairs requires additional information. In the absence of genotype‐to‐phenotype mapping, consistent patterns of divergence across the landscape are suggestive of ecological species (Nosil, 2012; Schluter, 2009), in essence using a genotype‐to‐environment association to infer adaptive divergence. If multiple, sympatric population pairs arrayed across a landscape demonstrate divergence in the same genomic regions, this suggests two possible scenarios: (a) repeated evolution of divergent adaptation between sympatric population pairs at local geographic sites, that could either be based on standing genetic variation or new mutations (e.g., Doellman et al., 2019; Feder, Berlocher, et al., 2003), or (b) a single adaptive divergence event, followed by geographic dispersal (e.g., Cook, Rokas, Pagal, & Stone, 2002). Although it may be difficult to distinguish between these two scenarios, both support adaptive divergence among populations.

The European cherry fruit fly, *Rhagoletis cerasi* (L., 1758) (Diptera: Tephritidae), is an important agricultural pest distributed throughout Europe and western Asia. This tephritid fly is responsible for considerable economic loss due to the damage it causes to cherry production (Boller, Haisch, & Prokopy, 1970). The ecology of *R. cerasi* is similar to that of *R. pomonella*. The fly has a univoltine life‐cycle with an annual winter pupal diapause period synchronising *R. cerasi* adult eclosion with the fruiting of its host plants, *Prunus* spp. (Rosaceae) and *Lonicera* spp. (Caprifoliaceae) (Boller & Bush, 1974; Schwarz, McPheron, Hartl, Boller, & Hoffmeister, 2003). Females oviposit a single egg in ripening fruit, and larval feeding causes physical damage that increases fruit susceptibility to fungal and bacterial infections (Boller & Prokopy, 1976). Larvae exit the fruit after feeding and enter a pupal diapause within the soil under the host plant until the following spring, when adults start to emerge again (Ozdem & Kilincer, 2005). Adult flies are poor dispersers, especially when host fruit is available, suggesting that fly populations may have high fidelity to particular host plants (Boller & Remund, 1983).

Previous studies on *R. cerasi* have characterised several traits that may contribute to host‐related genetic differentiation and race formation. Unlike *R. pomonella*, seasonal fruiting time differences between *Prunus* and *Lonicera* host plants does not appear to drive strong differences in fly phenology, although slight differences in eclosion times between *Prunus‐* and *Lonicera*‐infesting flies exist (Bakovic, Stauffer, & Schuler, 2018; Boller & Bush, 1974). Boller, Katsoyannos, and Hippe (1998) showed that exposure of mature female flies to a particular host fruit increased oviposition preference for that fruit. This is expected to increase host fidelity because young flies that eclose underneath the tree they were feeding on as larvae will most likely first encounter their natal tree, which will increase their propensity to subsequently use the natal host for oviposition. Other differences not tied to the natal environment have been observed, such as lowered host marking pheromone discrimination in *Lonicera* as compared to *Prunus* flies, a finding attributed to differential learning behaviours (Boller & Aluja, 1992). In addition, the timing of endosymbiont acquisition differs between *Prunus* and *Lonicera* flies. *Wolbachia*, a maternally inherited endosymbiont, which causes unidirectional incompatibilities between infected males and uninfected females of *R. cerasi*, reached fixation in *Prunus* flies, while only infecting 17% of *Lonicera* flies in German populations (Schuler et al., 2016). These results provide some evidence of phenotypic differentiation among host‐associated flies and suggest that mating may not be entirely random.

Previous genetic surveys based on a limited number of markers have provided a cursory understanding of the population structure of *R. cerasi*. An analysis of 13 cross‐amplified microsatellite loci for flies across Europe suggested the existence of spatial genetic structuring among populations collected at geographic distances ranging from 180 to 1,500 km (Augustinos et al., 2014). This geographic variation could reflect the colonisation history of *R. cerasi*, which is hypothesised to have migrated to Europe from western Asia (Fimiani, 1989), presumably following its *Prunus avium* host. However, ancient pollen data from *Prunus* host plants is lacking, making it difficult to confirm the colonization hypothesis. Nevertheless, there are reasons to suspect that *R. cerasi* should display geographic structure aside from its potential colonization of Europe. As discussed above, *R. cerasi* has low adult mobility (Boller & Remund, 1983), presumably facilitating local differentiation. In this regard, the diapause intensity of *Prunus*‐infesting flies has been shown to differ between German and Greek populations, suggesting an adaptive response to differences in phenology patterns of regional cherry cultivars (Moraiti, Nakas, & Papadopoulos, 2014, 2017). Additionally, the regulation of carbohydrate and glycogen reserves during diapause differs between highland and coastal populations of cherry‐infesting flies in Greece (Papanastasiou, Nestel, Diamantidis, Nakas, & Papadopoulos, 2011). To the extent that these differences are genetically‐based, they should affect the structure of local and regional populations of *R. cerasi*.

In addition to geographic differentiation among *R. cerasi* populations, there is limited evidence for host‐related genetic divergence. Schwarz et al. (2003) compared three polymorphic allozyme loci and found that one locus, Mannose phosphate isomerase (*Mpi)*, showed significant, although modest, allele frequency differences between *Prunus‐* and *Lonicera*‐infesting flies (all three loci: *F*_CT_ = 0.006, *p* = .04; *Mpi*: *F*_CT_ = 0.025, *p* < .01) after accounting for the effects of geographic distance. This same locus has also been shown to be differentiated between the ancestral hawthorn‐ and derived apple‐infesting host races of *R. pomonella* (Feder, Chilcote, & Bush, 1990a; McPheron, 1990). Schuler et al. (2016), however, found no evidence for host‐related differentiation or population structure in *R. cerasi* based on 13 species‐specific microsatellites. Thus, whether *R. cerasi* is a panmictic generalist in Europe or whether it forms locally host‐adapted populations remains an open question. To resolve these issues, we incorporated the repeated sampling of sympatric host‐associated populations and performed a genome‐wide survey of geographic and host‐related genetic differentiation for *R. cerasi*. Flies were sampled across Europe and Iran, including four sympatric *Prunus‐Lonicera* sites. Some of the specimens analysed here were also genotyped for microsatellites in the study of Schuler et al. (2016), allowing for comparisons between results obtained using different types of markers.

## 2 MATERIALS AND METHODS

### 2.1 Samples and DNA material

**Figure 1:**
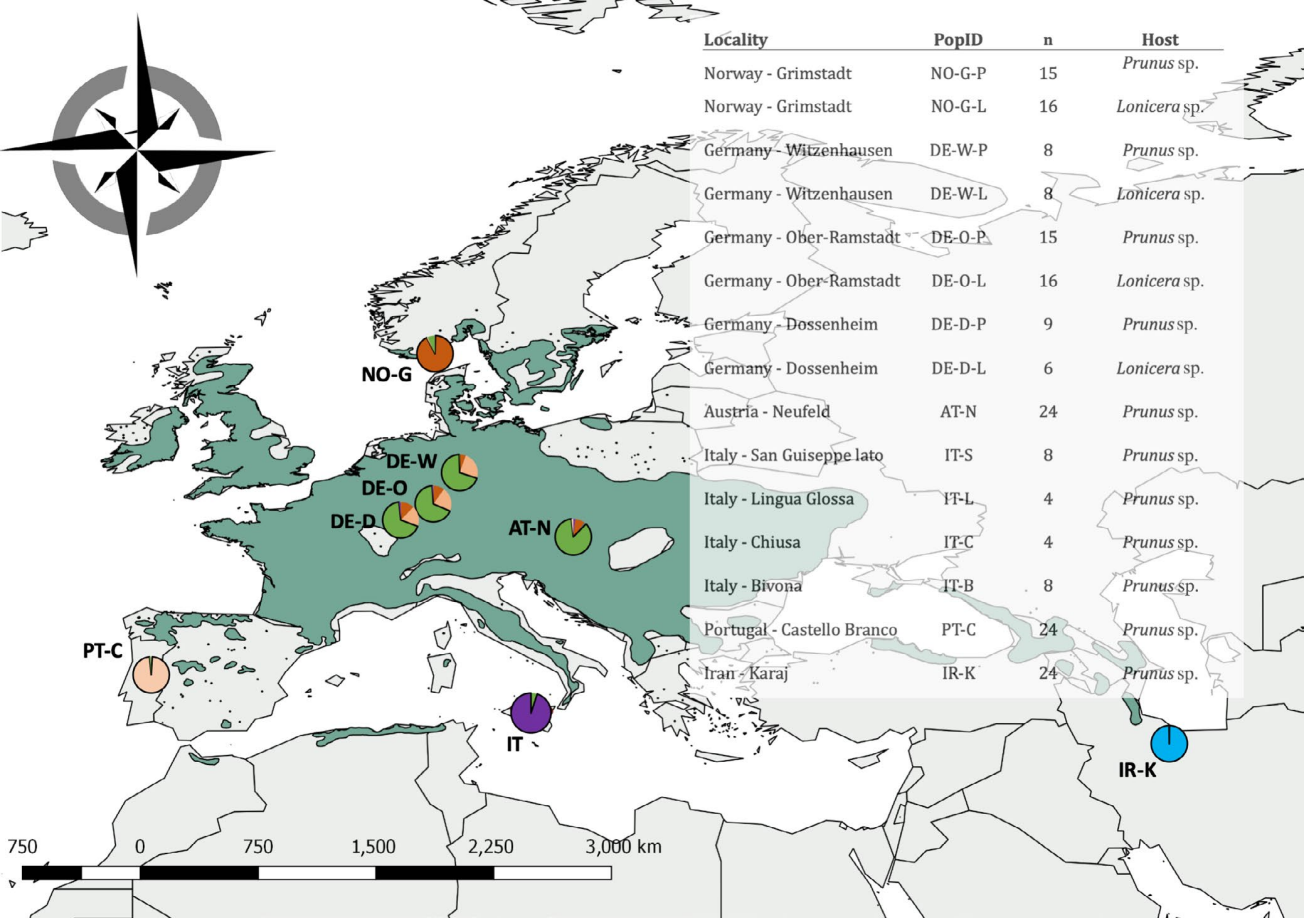
Map of study area including 15 *Rhagoletis cerasi* sampling locations throughout Europe and Iran. Coloured pie charts represent sampling sites included in this study with colours corresponding to Structure analysis. Table within figure includes locations of sampling sites, population IDs used throughout this study, numbers of individuals sampled (*n*) and host plant from which fly samples were collected. Dark green surface represents the geographic range of the host plant *Prunus avium* [Colour figure can be viewed at http://wileyonlinelibrary.com]

A total of 192 *Rhagoletis cerasi* individuals were genotyped from specimens collected as larvae in infested cherry or honeysuckle fruit at eleven localities across Europe and Iran between 2000 and 2009 (Figure 1; Tables S1 and S2). The localities included sites in Norway (NO), Germany (DE), Austria (AT), Portugal (PT), Italy (IT) and Iran (IR) (Figure 1). The widespread distribution of collection sites allowed for investigation of the effects of long geographic distance and geomorphic barriers on the genetic structure of *R. cerasi* populations. Sympatric collections were made at four sites (one in NO and three in DE) where *Prunus* spp. and *Lonicera* spp. host plants co‐occurred and were separated by distances of 0.9 to 3.2 km (Figure 1, Tables S1 and S2). Out of our 192 sampled individuals, previous DNA extracts from 147 specimens (Arthofer et al., 2009; Schuler et al., 2016) were reanalysed, while DNA from an additional 45 pupae was extracted using the GenElute Mammalian Genomic DNA miniprep kit (Sigma Aldrich, St. Louis, MO) following the manufacturer's instructions. DNA concentrations for all samples were estimated using a NanoDrop 2000C (Thermo Fisher Scientific, Waltham, MA) and were standardised to range between 30 and 60 ng/µl by either dilution or evaporation prior to library preparation. After sequencing, three individuals were discarded from further analysis due to lower coverage and quality of reads (see below), leaving a total of 189 specimens.

### 2.2 Library preparation and sequencing

We used genomic DNA from *R. cerasi* to generate a reduced representation library using a modified version of the Peterson, Weber, Kay, Fisher, and Hoekstra (2012) double digest restriction associated DNA sequencing (ddRADSeq) approach (Parchman et al., 2012). Briefly, 200 ng of genomic DNA per sample were digested using the restriction endonucleases *Eco*RI and *Mse*I. Barcoded *Eco*RI and nonbarcoded *Mse*I adapters, containing forward and reverse Illumina sequencing primers, respectively, were ligated to the digested DNA fragments. This step provided a unique barcode sequence for each individual fly. Digested and adaptor‐ligated fragments were PCR amplified using primers complementary to the *Eco*RI and *Mse*I adapters. PCR products of all samples were first pooled and then purified using magnetic beads (Agencourt AMPure XP, Beckman Coulter, Indianapolis, IN). A BluePippin System (automated DNA size selection system, Sage Science, Beverly, MA) was then used to size‐select fragments 400–500 bp in length from the pooled and purified PCR products. Final fragment size range of the library was assessed using a Bioanalyser 2100 (Agilent Technologies, Santa Clara, CA) and the DNA concentration of the library was measured on a Qubit 2.0 Fluorometer (Invitrogen, Grand Island, NY). Finally, the library containing all 192 samples was 100 bp paired‐end sequenced on a single lane of an Illumina HiSeq2000 machine (BGI Americas, Cambridge, MA).

### 2.3 Data processing

Data processing and analyses were performed on the Vienna Scientific Cluster, VSC3. Raw sequencing output resulted in 273,988,021 raw 100 bp paired‐end reads (233,473,324 demultiplexed reads). Because this study relied on de novo fragment assembly, only forward reads were included in all analyses described below. In brief, we generated consensus sequences de novo using stacks version 1.4 (Catchen, Hohenlohe, Bassham, Amores, & Cresko, 2013) and mapped our reads to this consensus using bwa version 0.7.12 (Li & Durbin, 2009). A total of 216,197,186 reads mapped to the consensus (93% of demultiplexed reads). SNP calling was performed using *GATK*'s UnifiedGenotyper version 3.4 (McKenna et al., 2010), resulting in 773,447 raw unfiltered SNPs. From these raw SNPs we generated two data sets, one in which the coverage for included loci was sufficient for analyses requiring called genotypes, and a second, larger set of SNPs that included additional lower coverage loci that could be analysed by accounting for genotype uncertainty using genotype likelihoods (Nielsen, Korneliussen, Albrechtsen, Li, & Wang, 2011). Both data sets were filtered for a minimum read depth of 50 (including all individuals) and a minimum sequencing quality of 21. To obtain genotype likelihoods, we removed: (a) all nonbiallelic SNPs (retaining 762,794 SNPs), (b) SNPs found in less than 90% of individuals sequenced (retaining 192,762 SNPs), (c) all SNPs that deviated from the expectation of equal allele counts among heterozygotes, by applying a binomial test and discarding all SNPs where the null hypothesis of *p* = .5 (*p* being the allele frequency of the more common allele) was rejected with *α* = .05 (O'Leary, Puritz, Willis, Hollenbeck, & Portnoy, 2018; Parchman et al., 2012) (retaining 123,444 SNPs), and (d) SNPs with rare allele frequencies <0.05 (minor allele frequency, MAF < 0.05) (retaining 40,040 SNPs). For genotype calls, we additionally filtered out all SNPs whose GQ (genotype quality) value was under 15 (retaining 4,973 SNPs) and further eliminated 859 SNPs showing Hardy‐Weinberg heterozygote excess (estimated in each population separately) to account for possible paralogs, retaining 4,114 SNPs. Finally, only the first SNP found on RAD contigs was used to minimize linkage bias. The first data set resulted in 23,964 per locus genotype likelihoods with an average read depth per individual of 4.85×, while the second resulted in 2,494 per locus genotype calls with an average read depth per individual of 9.74× (see Tables S3 and S4). When subsetting the data to run analyses on only sympatric *Prunus*‐*Lonicera* flies, we removed additional nonpolymorphic sites, which we address in the following sections below.

### 2.4 Population genetic parameter estimates and isolation‐by‐distance

The second data set with 2,494 SNPs was used to calculate population genomic summary statistics (expected heterozygosity *H*_e_, observed heterozygosity *H*_o_, nucleotide diversity *π* and inbreeding coefficient *F*_IS_) and average pairwise *F*_ST_ values among geographic sites and between host plants, based on the populations module in stacks (calculated using the AMOVA *F*_ST_ estimate with the bootstrap_fst option). Confidence intervals (0.95) were calculated from individual *F*_ST_ and *F*_IS_ values for each SNP for each population using the groupwiseMean function of the rcompanion package (Mangiafico, 2017) in r version 3.1.4 (R Core Team, 2014). Significance for nucleotide diversity (π) differences among populations was first assessed by conducting a one‐way ANOVA. As part of a post‐hoc analysis, populations were then assigned significance groupings using Tukey's honestly significant difference tests within the agricolae r package (Mendiburu, 2014). To test for isolation‐by‐distance (IBD), we conducted a simple Mantel test by correlating population pairwise *F*_ST_ values to pairwise geographic distances (pairwise geographic distances are provided in Table S2).

### 2.5 Genetic clustering by geography

The 2,494 SNP data set was also used to asses population structure by three different methods. First, a Bayesian approach as implemented in structure version 2.3.4 (Pritchard, Stephens, & Donnelly, 2000) was used to estimate the number of genetic clusters within our data. We used the admixture model implemented in structure and ran 15 replicates for each *K* value (number of subpopulations) assessed from *K* = 2 to *K* = 15, with a burnin period of 100,000 and 100,000 Markov Chain Monte Carlo (MCMC) repetitions after initial burnin. To estimate the number of clusters within our data, we used the delta *K* approach described by Evanno, Regnaut, and Goudet (2005), using structure harvester (Earl, 2012). Replicate runs with Bayesian methods commonly result in slightly different solutions (Jakobsson & Rosenberg, 2007). Therefore, the output from the 15 replicates of the best *K* value were aligned using the LargeKGreedy model in clummp version 1.1.2 (Jakobsson & Rosenberg, 2007), with 1,000 random input orders taken before calculating mean values. The program distruct version 1.1 (Rosenberg, 2004) was used to generate graphical output.

Second, principal component analysis (PCA) was performed using the R package adegenet with the function dudi.pc (Jombart, 2008; Jombart & Ahmed, 2011). The scaleGen function was used to scale allele frequencies to mean zero, with missing genotypes replaced by mean allele frequency values among individuals.

Third, to complement to the structure plots and PCA, we also used *R. cerasi* SNP data to generate phylogenetic trees. A bootstrapped maximum‐likelihood tree, with individual samples at its tips, was constructed using the SNPhylo pipeline with MAF < 0.05 and 1,000 bootstrap replicates (Lee, Guo, Wang, Kim, & Paterson, 2014).

### 2.6 Genetic clustering by host plant

Because we found that geographic variation among sites was much greater than that between host plants (see Section 3), a subset of individuals, representing the four *Prunus‐Lonicera* sympatric sites, was used to analyse genome‐wide differentiation between *Prunus* and *Lonicera* flies (Figure 1). All analyses testing host race formation in all four sympatric sites using genotype calls were run on 2,336 SNPs, as 158 additional SNPs were found to be monomorphic after subsetting the data set to include only the four sympatric *Prunus‐Lonicera* sites. First, we used a partial Mantel test to evaluate the correlation between *F*_ST_ values and host plant of origin, while controlling for geographic distance. The significance of the partial Mantel test was determined by comparing the partial correlation coefficient with a null distribution, which was generated by randomising the rows and columns of the first matrix, while keeping the other two constant and recalculating Pearson's correlation coefficients. Null distributions were generated by repeating the randomisation 10,000 times using the vegan package in r (Oksanen et al., 2018). All *F*_ST_ values were calculated using the populations module of stacks, as described above. Taking into account the ongoing debate about the most appropriate metric for distance matrix correlations (Bohonak, 2002), we used both raw and log‐transformed distance matrices. The precision of the partial Mantel test has been questioned for its lack of power and high type I error rate (e.g., Raufaste & Rousset, 2001). However, partial Mantel tests have been found to perform well under a variety of conditions, including using normally or non‐normally distributed data, and continuous or categorical data (e.g., Funk, Egan, & Nosil, 2011). We complement the partial Mantel test with several analyses based on allele frequency differences between the host ecotypes in the four sympatric sites before evaluating the influence of host plant affiliation on genetic divergence in *R. cerasi*.

To assess the genetic relationship between *Prunus* and *Lonicera* flies from the four sympatric sites, we constructed a neighbour joining network based on Nei's genetic distances, using the packages poppr (Kamvar, Tabima, & Grünwald, 2014) and ape (Paradis, Claude, & Strimmer, 2004) in r. We also investigated the pattern of host‐related genetic differentiation at each of the four sympatric sites through discriminant analysis of principal components (DAPC), using the r package adegenet. Values of mean probabilities for correct assignment of individuals to populations in DAPC were compared with simulated values (*n* = 10,000 replicates) to determine if they exceeded the upper 0.95 quantile of the randomised distributions. Randomisations were computed by randomly shuffling the host population labels and running DAPC. Finally, we ran DAPC on all four sympatric pairs of host populations (*K* = 8) to compare levels of geographic vs. host‐related clustering (see run details in Table S4). The advantage of DAPC is that this method works well with structured and nonparametric data and allows extraction of the highest contributing SNPs to the axes of greatest differentiation between groups.

### 2.7 Consistency of *Prunus‐Lonicera* fly divergence across geography

To examine the degree to which the same loci contributed to host‐related differentiation across sites, we calculated and compared correlations of local SNP allele frequency differences between *Prunus* and *Lonicera* flies across pairs of sympatric sites. Correlations were calculated for four different SNP sets representing: (a) all loci, (b) only loci showing significant (0.05; method see below) allele frequency differences between *Prunus* and *Lonicera* flies at both geographic sites being compared, (c) only loci showing significant allele frequency differences between *Prunus* and *Lonicera* flies in at least three of the four total sympatric sites, and (d) loci showing significant allele frequency differences between *Prunus* and *Lonicera* flies at all four sympatric sites.

SNP allele frequencies for *Prunus* and *Lonicara* flies at each site were estimated for the more inclusive, low coverage set of 23,964 SNPs (see above) using genotype probabilities as:

$$P=\;\frac{\sum_{i=1}^{n}\sum_{j=1}^{3}\left(g_{j}\times p\left(g_{j}|D_{i}\right)\right)}{2\times n}$$

where *n* = number of individuals, *g*_1:3_ = [0,1,2] (number of alternate allele copies in a homozygote, heterozygote and alternate homozygote, respectively), and *p*(*g_j_*|*D_i_*) is the probability of genotype *j* given the sequence data *D* at locus *i*, as estimated in *GATK* (Gompert et al., 2012; Parchman et al., 2012). Using genotype likelihoods allowed for the use of a much broader portion of the read coverage distribution (Nielsen et al., 2011). We used a permutation test to determine significance for differences in allele frequencies between groups of individuals sampled from different host plants, randomly shuffling the host plant label and calculating allele frequency differences 10,000 times to generate null distributions. Estimated allele frequency differences falling above the 0.975 or below the 0.025 quantile were considered statistically significant. We used these significance assignments only to subset the data for estimating correlations in allele frequency differences across sites (see above), and, thus, did not use multiple testing corrections. However, we did use the significance of allele frequency differences in conjunction with other evidence to identify candidate loci associated with host plant differences (see below).

To test whether SNPs exhibiting significant allele frequency differences are shared among the four sympatric sites more than expected by chance, we performed a Monte Carlo permutation test that accounts for nonindependence among loci. In each permutation, individuals were randomly assigned to a host plant within each sympatric site (preserving LD relationships among loci), and the statistical significance of allele frequency differences between hosts was estimated as described above. Then numbers of significant loci shared among geographic sites were calculated. We performed 10,000 permutations to generate a null distribution of counts of shared loci.

### 2.8 SNPs associated with host plant

bayenv2 (Coop, Witonsky, Di Rienzo, & Pritchard, 2010) was used on the 2,336 called SNPs for the four sympatric populations to identify individual SNPs associated with *Prunus* vs. *Lonicera* host plants. First, we used 100,000 iterations sampling every 500 iterations to generate a covariance matrix among the sympatric *Lonicera* and *Prunus* pairs at the four geographic sites. An average of all sampled covariance matrices was used for the actual association test. We ran the association test with 100,000 iterations and a single environmental variable (host plant), where *Prunus* = 1 and *Lonicera* = 0. The program produces Bayes factors for each SNP, which represent a ratio between support for H1 (a SNP is associated with the environmental variable) and support for H0 (SNP is not associated with the environmental variable), and Spearman's rho values, which represents the magnitude and direction of the association. We selected the top 1% of SNPs that contained the highest Bayes factors conditioned that they were also within the top 10% of rho values as candidate loci.

As flies tended to cluster according to host affiliation within Germany, but according to geography when comparing Norwegian flies (see Section 3) we ran independent bayenv2 runs on the German and Norwegian populations to test whether the same outlier SNPs are associated with host plant in both these regions. bayenv2 was run as described above except on 2,294 SNPs in Germany and 2,094 SNPs in Norway, due to the exclusion of monomorphic markers after subsetting the data.

### 2.9 Linkage disequilibrium

Sets of loci under divergent selection may exhibit elevated linkage disequilibrium (LD) through either physical linkage (e.g., loci co‐localise on chromosomal inversions or other linkage blocks; Comeault, Carvalho, Dennis, Soria‐Carrasco, & Nosil, 2016; Feder, Roethele, Filchak, Niedbalski, & Romero‐Severson, 2003), or correlational selection (Korunes & Noor, 2017; Sinervo & Svensson, 2002; Yeaman, 2013). To test whether outlier SNPs were in higher LD with each other than background LD levels for the 2,336 SNP data set, we calculated LD separately in populations at each of the four sympatric sites for: (a) *Lonicera* flies, (b) *Prunus* flies, and (c) *Prunus* and *Lonicera* flies pooled together. Only those SNPs previously identified as being associated with host plant in at least two of the three analyses performed (allele frequency differences, DAPC, and bayenv2; *n* = 17) were included in LD calculations. We also calculated the mean LD for all 2,336 called SNPs. To test whether candidate loci displayed higher LD than the background LD of all SNPs, a permutation test was conducted by randomly drawing 17 (out of 2,336) SNPs and calculating LD 1,000,000 times. This step was repeated for each host plant separately and for *Prunus* and *Lonicera* populations pooled together at each site. The LD.measures command from the ldcorsv r package (Desrousseaux, Sandron, Siberchicot, Cierco‐Ayrolles, & Mangin, 2017) was used to calculate *r* ^2^ values between SNPs as the metric for LD.

## 3 RESULTS

### 3.1 Population genetic parameter estimates and isolation‐by‐distance

Higher observed than expected heterozygosity was seen at all geographic sampling sites, whereas *F*_IS_ values were either not significantly different from zero or were close to zero at all locations (Table 1). German and Austrian sites had the highest nucleotide diversity (*π*) compared to those in Iran, Portugal, Italy or Norway (Table 1). Pairwise *F*_ST_ values between sites were all relatively low, ranging from 0.004 to 0.143 (Table S5) and were positively related to geographic distance between sites, supporting a pattern of IBD (*r* = .94, *p* < 2.2e−16, Mantel test; Table S5 and Figure S1).

**Table 1:** Population genetic summary statistics for 15 *Rhagoletis cerasi* sampling sites used in this study showing nucleotide diversity (*π*), observed (Obs. Het.) and expected heterozygosity (Exp. Het.) and the inbreeding coefficient along with 95% confidence intervals. Calculations were made using 2,494 SNPs

Pop ID	Obs. Het.	Exp. Het.	*π*	*F*_IS_	95% CI *F*_IS_
NO‐G‐P	0.25	0.2423	0.2518^b^	0.0021	–0.00611/0.0104
NO‐G‐L	0.2559	0.2465	0.2556^b^	–0.0019	–0.00984/0.00604
DE‐W‐P	0.2618	0.2379	0.2576^b^	–0.0068	–0.017/0.00338
DE‐W‐L	0.2786	0.2455	0.2685^ab^	–0.0178	–0.0287/–0.00693
DE‐O‐P	0.2709	0.2478	0.2589^b^	–0.0294	–0.0377/–0.021
DE‐O‐L	0.2746	0.2578	0.2686^ab^	–0.0159	–0.0243/–0.00747
DE‐D‐P	0.3018	0.2571	0.2821^a^	–0.041	–0.0517/–0.0303
DE‐D‐L	0.2725	0.2354	0.2661^ab^	–0.0115	–0.0226/–0.00045
AT‐N‐P	0.2725	0.2562	0.2633^ab^	–0.0208	–0.028/–0.0135
IT‐S‐P	0.2327	0.2088	0.2266^c^	–0.0109	–0.0208/–0.000887
IT‐L‐P	0.2266	0.1911	0.2275^c^	0.0015	–0.00918/0.0123
IT‐C‐P	0.2099	0.1792	0.2121^c^	0.0036	–0.00714/0.0144
IT‐B‐P	0.2344	0.2032	0.2219^c^	–0.0254	–0.0351/–0.0156
PT‐C‐P	0.2402	0.2242	0.2313^c^	–0.02	–0.0271/–0.013
IR‐K‐P	0.2148	0.2084	0.2141^c^	–0.0004	–0.00697/0.0062

Letters in superscript denote which groups are significantly different as determined by TukeyHSD. For collection site abbreviations, see Figure 1.

### 3.2 Geographic genetic clustering of populations

**Figure 2:**
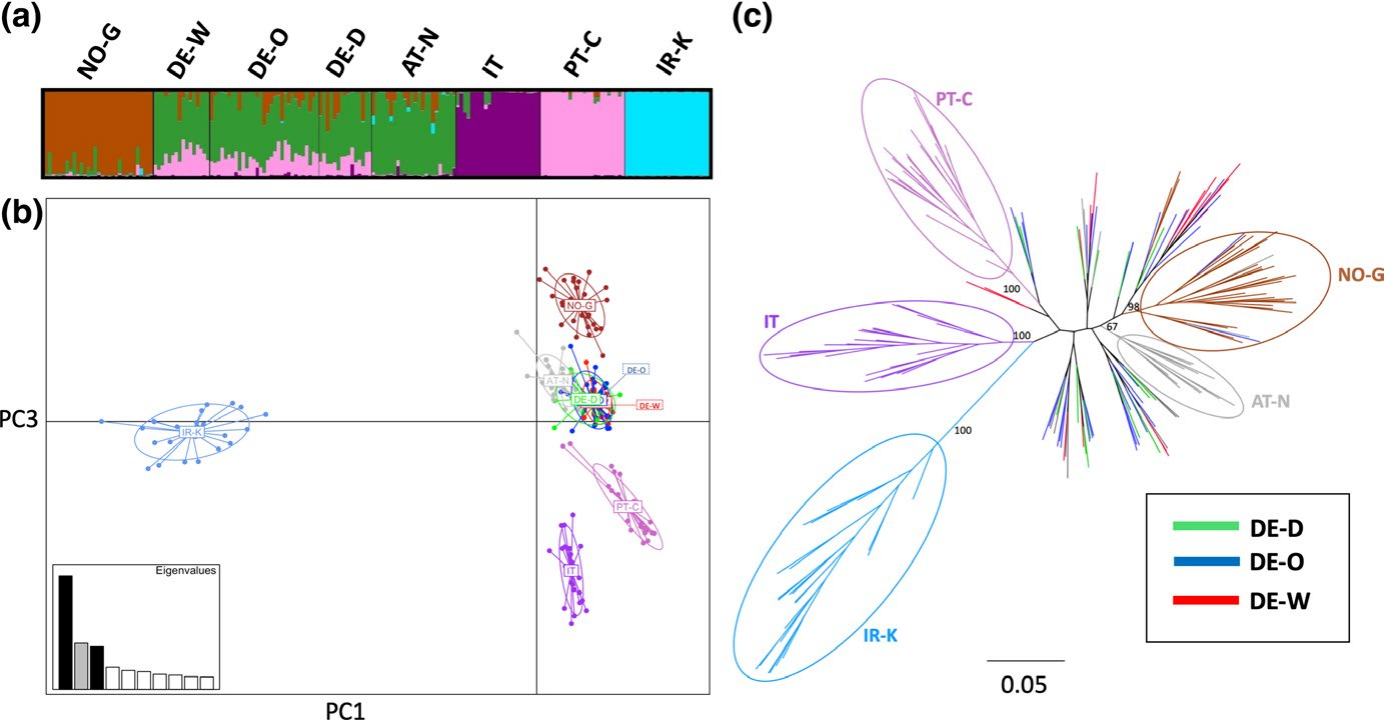
Population structure of *Rhagoletis cerasi* samples across geography inferred using called genotypes at 2,494 SNPs. (a) *structure* plot depicting individual membership probabilities at a *K* = 5. Individuals are ordered according to geographic site. (b) Clustering relationships among geographic sites based on PCs calculated from the same SNP data set, using PC 1 and PC 3, which explain 8.27% and 3.23%, of the variance, respectively. (c) Bootstrapped maximum likelihood tree with individual samples at its tips. For collection site abbreviations, see Figure 1 [Colour figure can be viewed at http://wileyonlinelibrary.com]

Bayesian cluster inference implemented in structure implied the existence of five different genetic clusters (*K* = 5) of flies largely defined geographically as: (a) Iran, (b) Portugal, (c) Italy, (d) Norway, and (e) the German and Austrian sites (Figure 2a). Although, *K* = 4 resulted in the highest delta *K*, we discuss *K* = 5 as the most biologically relevant, as it distinguishes the sites by geography (Figure 2a; see different *K* values in Figure S2). Individuals sampled from Germany formed a cohesive cluster under all *K* values (Figure S2). Additionally, there was some evidence for shared ancestral polymorphism or recent admixture between German sites and Austrian, Portuguese and Norwegian sites.

The same general pattern of geographic genetic differentiation was observed in the PCA, with Iran, Austria, Germany, Portugal, Italy and Norway forming separate clusters (Figure 2b). In addition, a PCA conducted only on the German and Austrian sites indicated a general lack of genetic structure within Germany but clear separation between the German and Austrian sites (Figure S3).

The ML phylogenetic tree further supported the clusters inferred from structure analysis and PCA. Most flies from Iran, Austria, Portugal, Norway, and Italy were on different and distinct branches from one another with high bootstrap support (Figure 2c). German flies, although genetically different from the other populations, did not form a distinct branch but were dispersed in varying positions in the tree (Figure 2c).

### 3.3 Evidence for host‐associated genetic divergence

The magnitude of host‐related genetic divergence was generally less than divergence among geographic sites (Table S5–*F*_ST_ values among geographic sites and flies affiliated to different host plants). Nevertheless, several lines of evidence supported the existence of significant host plant‐related genetic differentiation between *Prunus* and *Lonicera* flies. First, a partial Mantel test indicated a significant effect of host plant on genetic differentiation between *Prunus* and *Lonicera* flies, while controlling for geographic variation (raw data: *r* = .274, *p* = .051; log‐transformed data: *r* = .456, *p* < .0018; Table S6).

**Figure 3:**
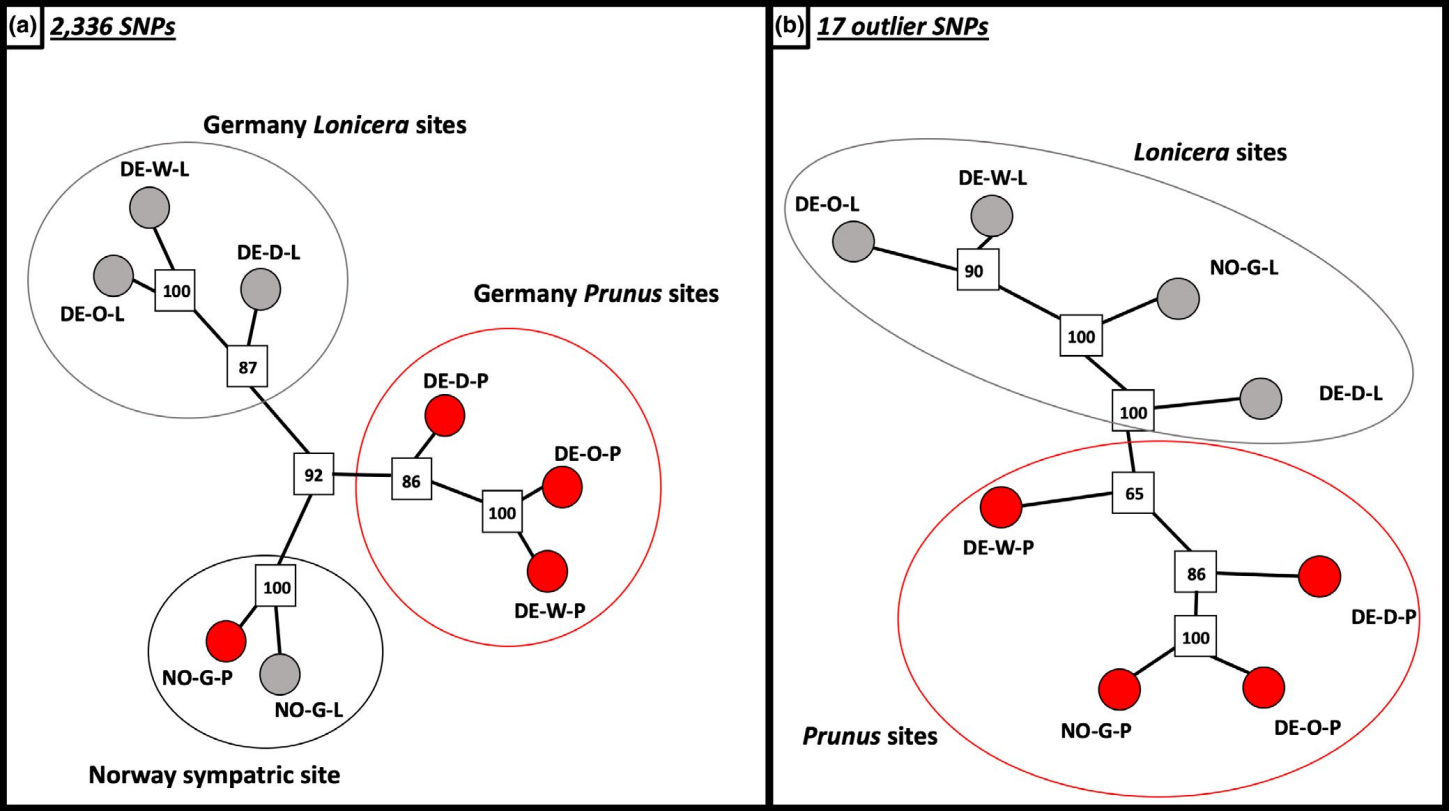
Unrooted neighbour‐joining network of four pairs of *Prunus*‐*Lonicera* sympatric *Rhagoletis cerasi* flies based on (a) 2,336 called SNPs and (b) 17 outlier SNPs. The network was generated using Nei's genetic distances and 1,000 bootstrap replicates (squares). The last letter of population labels indicates host plant of origin; P, *Prunus*; L, *Lonicera* [Colour figure can be viewed at http://wileyonlinelibrary.com]

Second, the NJ network, based on all 2,336 called SNPs, indicated that groups of sampled flies clustered genetically by host association, though such clustering varied depending on geographic distance. All flies collected in Germany (within roughly 250 km) clustered by host plant rather than by geographic location (Figure 3). In contrast, flies collected from both host plants at the Norwegian site (approximately 1,000 km from the German sites) genetically clustered together, rather than with the host‐associated clusters identified in Germany. Thus, genetic clustering by host plant is subsumed within a larger geographic pattern of variation.

**Figure 4:**
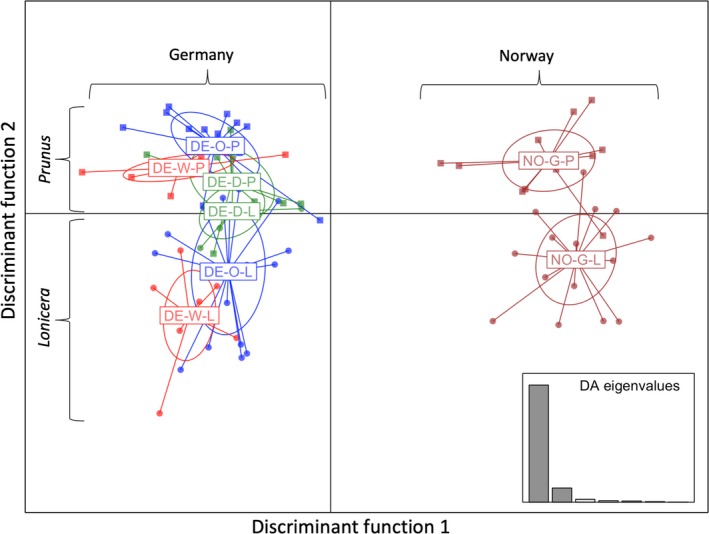
Discriminant analysis of principal components plot of scores of individual *Rhagoletis cerasi* flies for discriminant functions 1 (85.24% variation explained) vs. 2 (10.33% variation explained) for individual *Prunus* and *Lonicera* flies from one sympatric site in Norway (NO) and three sympatric sites in Germany (DE). All population labels ending with a letter “L”, represent *Lonicera* flies and population labels ending with a letter “P”, represent *Prunus* flies. For additional clarity, *Prunus* flies are represented by squares, while *Lonicera* flies by circles. The inset plot shows a histogram of DA eigenvalues, providing a relative measure of the ratio of geography vs. host plant variation explained by discriminant functions 1 and 2 for the PCs retained (13) in the DAPC analysis [Colour figure can be viewed at http://wileyonlinelibrary.com]

**Figure 5:**
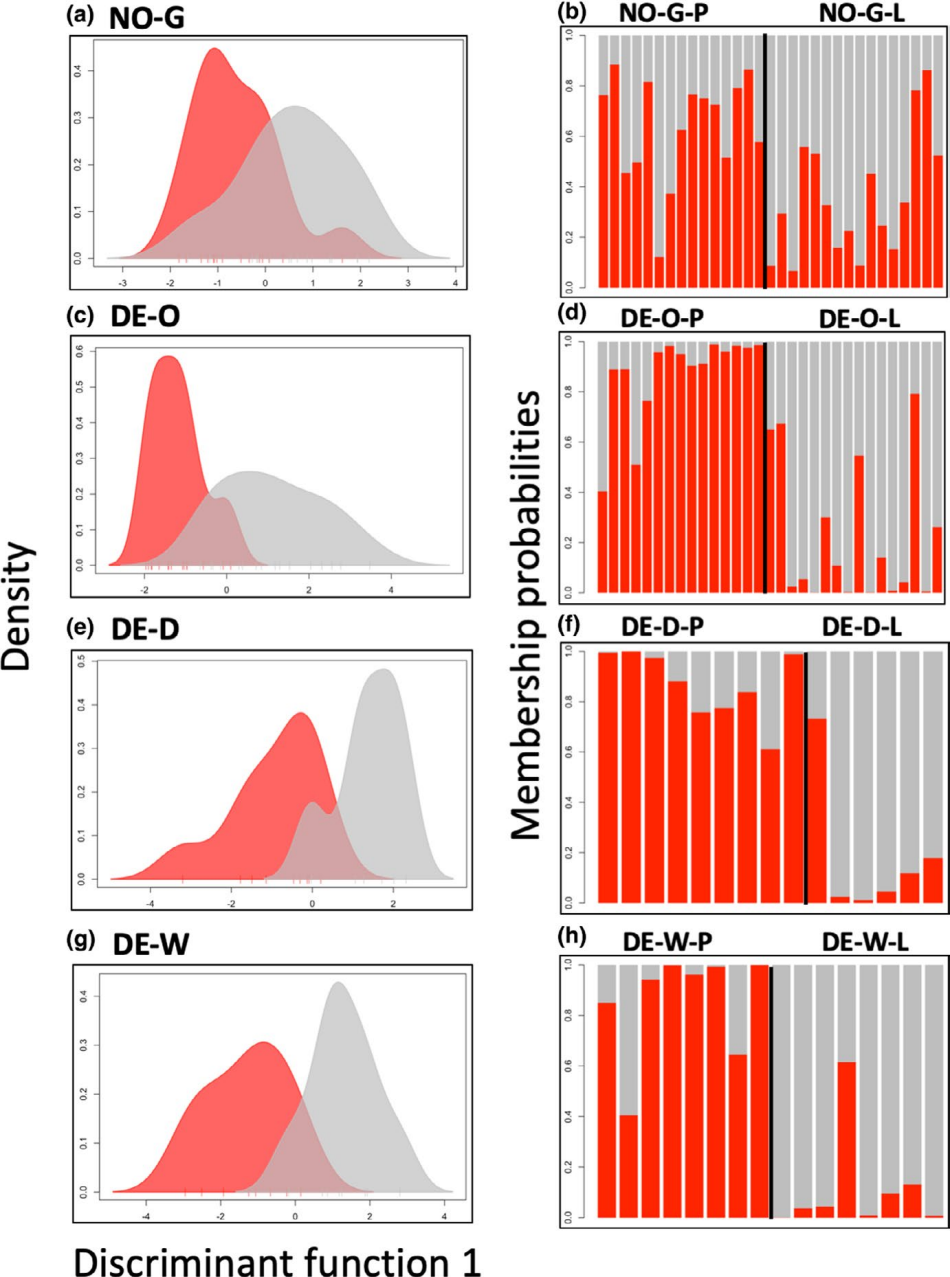
Discriminant analysis of principal components partially discriminates *Prunus*‐ and *Lonicera*‐associated *Rhagoletis cerasi* flies. The first column (a, c, e, g) displays kernel density plots illustrating the distribution of individual discriminant function 1 scores for flies sampled from *Prunus* (red) and *Lonicera* (grey) hosts. The second column (b, d, f, h) displays assignment probabilities to *Prunus* and *Lonicera* (again, red and grey, respectively). The rows represent different geographic sites. Number of retained principal components (PCs), mean assignment probabilities to the correct host (AP), and *p*‐value testing whether assignment probabilities were greater than expected by chance (*P*) were as follows: (a,b) Norway, Grimstadt (NO‐G), PCs = 1, AP = 0.634 ± 0.056 *SE*, *p* = .005; (c,d) Germany, Ober‐Ramstadt (DE‐O), PCs = 1, AP = 0.791 ± 0.056 *SE*, *p* < 1e−4; (e,f) Germany, Dossenheim (DE‐D), PCs = 3, AP = 0.771 ± 0.088 *SE*, *p* = .018; (g,h) Germany, Witzenhausen (DE‐W), PCs = 1, AP = 0.638 ± 0.056 *SE*, *p* = .003 [Colour figure can be viewed at http://wileyonlinelibrary.com]

Third, DAPC including only the four pairs of *Prunus‐Lonicera* sympatric sites revealed a clear axis of genetic variation separating *Prunus*‐ from *Lonicera*‐associated individuals. Though the first discriminant function (85.24% of the variation explained) separated flies by geography (Norway vs. Germany), the second discriminant function (10.33% of the variation explained) separated flies by host‐affiliation (Figure 4). However, the separation between flies collected on the two host plants was not complete. This is further illustrated by Figure 5, showing that although mean DAPC assignment probabilities to the correct host within each site separately were more accurate than expected by chance, values ranged from 0.634 ± 0.056 (Grimstadt, Norway; *p* = .001) to 0.791 ± 0.056 *SE* (Ober‐Ramstadt, Germany; *p* < .0001). It is important to note that assignment probabilities and the identification of host plant‐associated SNPs were conducted in separate DAPC analyses. When running DAPC on all four sympatric sites, the first discriminant function reflects geography, while when running DAPC on each sympatric site separately the first discriminant function reflects host association.

**Figure 6:**
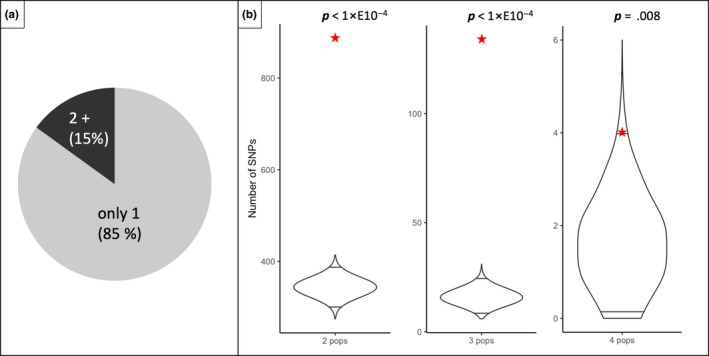
Pie chart and violin plots representing shared and nonshared host adaptation in four sympatric *Prunus‐Lonicera Rhagoletis cerasi* flies. (a) comparison of number of differentiated SNPs found only within one site with number of differentiated SNPs shared by more than one site. (b) Nevertheless, within this minority, a significant amount of shared differentiation is found by looking at comparisons of observed numbers of shared SNPs (red stars) with simulated distributions (violins) of differentiated SNPs that are found in any two, three and all four population pairs. Horizontal lines within the violins are the 0.025 and 0.975 quantiles [Colour figure can be viewed at http://wileyonlinelibrary.com]

Fourth, the general pattern of host‐related genetic differentiation was remarkably consistent across geographic sites. The mean pairwise correlation of allele frequency differences between *Prunus* and *Lonicera* flies for all 23,964 SNPs across the four sympatric field sites was *r* = .113 (*n* = 6 pairwise correlations with *r* values ranging from .047 to .202; all significantly different from zero at *p* < 2e−13; Table 2). However, correlations calculated using subsets of SNPs significantly associated with host plants in two or more populations yielded substantially higher correlations with *r* values ranging from .298 to .986 (all significantly different from zero at *p* < 2e−4 except in the case of 4 SNPs shared in all four sympatric sites, where correlations were not significant; Table 2). The directionality of allele frequency differences between hosts was also consistent across geographic sites, though only for SNPs whose allele frequencies consistently displayed significant differences between *Prunus* and *Lonicera* flies. Allele frequency differences were on average in the same direction 74% (range 56.76%–87.42%), 84% (range 74.63%– 92.54%) and 88% (range 75%–100%) of the time for SNPs displaying significant host‐associated variation in both populations being compared, and in at least three and all four sympatric sites, respectively (Table 2). Finally, the identity of SNPs displaying host‐associated differentiation was also consistent across sites. Though most significant SNPs were unique to a given sympatric comparison (85%), there was a significant excess of shared SNPs (compared to chance expectations) significantly associated with host across geographic sites, with observed values falling well outside the range of the null distributions (Figure 6).

**Table 2:** Correlations (*r* values) of allele frequency differences between pairs of sympatric *Prunus‐* and *Lonicera‐*infesting flies of *Rhagoletis cerasi* sampled across four sympatric sites in Europe; one in Norway (NO) and three in Germany (DE). Furthermore, percentages of times that allele frequency differences for SNPs between sympatric *Prunus* and *Lonicera* fly populations are in the same direction between sites is provided (% same)

	All SNPs (23,964)	0.05 in both populations	0.05 in at least three populations (134)	0.05 in four populations (4)	All SNPs (23,964)	0.05 in both populations	0.05 in at least three populations (134)	0.05 in four populations (4)
	R				% same			
(NO‐G)–(DE‐W)	0.131	0.732 (232)	0.864	0.371	51.235	82.328	91.791	100
(NO‐G)–(DE‐O)	0.148	0.765 (228)	0.888	0.401	52.449	85.526	92.537	100
(NO‐G)–(DE‐D)	0.080	0.481 (170)	0.382	–0.214	50.405	72.941	75.373	75
(DE‐W)–(DE‐O)	0.202	0.813 (334)	0.916	0.986	51.235	87.425	92.537	100
(DE‐W)–(DE‐D)	0.072	0.329 (111)	0.478	0.298	51.235	56.757	78.358	75
(DE‐O)–(DE‐D)	0.047	0.340 (92)	0.456	0.427	50.497	61.957	74.627	75

Correlations were performed (1) for all 23,964 SNPs, (2) for subsets of SNPs showing significant host‐related differences at both of the sympatric sites being compared, (3) for a subset of SNPs showing significant allele frequency differences in at least three of the four sympatric comparisons at sites, and (4) four SNPs found to be significantly differentiated in all four sympatric comparisons. Significance was estimated using a Markov chain Monte Carlo approach with 10,000 randomisations. Numbers in parentheses indicate the number of SNPs used in the comparison.

### 3.4 Candidate SNPs associated with host plants

**Figure 7:**
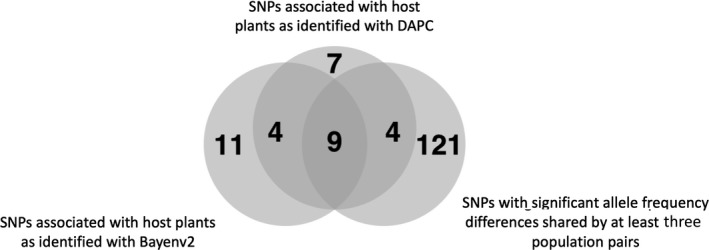
Venn diagram showing overlap of SNPs differentiating the host races found using three different analyses; *bayenv2* (*n* = 24)*,* SNPs showing significant allele frequency differences in at least three of the four sympatric *Prunus*‐*Lonicera* sites (*n* = 134), highest contributing SNPs to discriminant function 2 of the host race DAPC analysis (*n* = 24). SNPs identified with at least two different analyses (*n* = 17) are considered putative candidate SNPs under host‐related divergent selection

Using *bayenv2*, we identified SNPs with the highest association to host plant affiliation across the sampled geographic range and extracted the top 1% (*n* = 24; Table S7). These SNPs were then compared to the top 1% (*n* = 24) of SNPs that loaded most strongly onto the host race discriminant function of the DAPC (Figure 4) and to the 134 SNPs with significantly different allele frequencies between host fruits in at least three different sites (Table 2). A total of 17 loci where found to overlap in at least two of these analyses, which we consider candidate host‐associated loci with the highest statistical support (Figure 7; SNP labels can be found in Table S8). Furthermore, we identified a single SNP displaying host plant‐related divergence in both Germany and Norway, with 23 SNPs being unique to Germany and 20 SNPs being unique to Norway (Table S7).

BLAST search queries of these 17 SNP‐containing RAD contigs against the NCBI data base (Nucleotide collection [nr/nt]) largely returned mRNAs for *Rhagoletis zephyria*, the closest relative to *Rhagoletis cerasi* whose sequences are currently in the data base. To optimize our BLAST search we additionally BLASTed these reads directly onto the *R. zephyria* genome, which in some cases resulted in better matches (Table S9). Due to the small number of SNPs queried, whose BLAST hits also included uncharacterized loci, no gene ontology terms were found enriched using the DAVID gene functional classification tool (Huang et al., 2007).

### 3.5 Linkage disequilibrium

Linkage disequilibrium was estimated using the higher coverage data set (*n* = 2,336 loci) separately both within and between *Prunus* and *Lonicera* flies at each of the four sympatric sites (Table 3). Mean *r* ^2^ values between host‐associated SNPs were significantly higher than the average background for all SNPs. Mean *r* ^2^ values for all SNPs ranged from 0.031 to 0.110, while mean *r* ^2^ for the 17‐host associated SNPs significant in at least two of the three analyses performed (see above) ranged from 0.213 to 0.548. Mean *r* ^2^ values for simulated null distributions were very similar to values observed for all SNPs, while observed LD values of host‐associated SNPs in most cases lay far outside the range of the null distribution (Table 3).

**Table 3:** Linkage disequilibrium comparisons between 2,336 SNPs and 17 host‐associated SNPs in *Rhagoletis cerasi* samples collected in four *Prunus‐Lonicera* sympatric sites (one in Norway, NO; three in Germany, DE)

Groups	Mean LD all SNPs	Mean LD outliers	Mean LD simulated	Range simulated LD	*p*‐value
NO‐G	0.031	0.371	0.031	0.002–0.107	<1e−06
NO‐G‐P	0.054	0.287	0.054	0.002–0.157	<1e−06
NO‐G‐L	0.054	0.358	0.054	0.002–0.160	<1e−06
DE‐O	0.042	0.440	0.042	0.005–0.123	<1e−06
DE‐O‐P	0.072	0.213	0.072	0.002–0.213	<2e−06
DE‐O‐L	0.072	0.387	0.072	0.006–0.194	<1e−06
DE‐D	0.086	0.377	0.086	0.005–0.247	<1e−06
DE‐D‐P	0.108	0.487	0.108	0.000–0.562	<2e−06
DE‐D‐L	0.110	0.536	0.110	0.000–1.000	<9e−05
DE‐W	0.071	0.548	0.071	0.007–0.192	<1e−06
DE‐W‐P	0.096	0.309	0.096	0.000–0.298	<1e−06
DE‐W‐L	0.099	0.369	0.099	0.000–0.354	<1e−06

Linkage disequilibrium (LD) was calculated for each fly race separately and together at each sampling site. Simulations were derived by calculating LD of 17 randomly chosen SNPs 1,000,000 times and all values reported are *r* ^2^.

## 4 DISCUSSION

In the current study, we used ddRADseq on natural *Rhagoletis cerasi* populations to uncover signatures of host‐associated differentiation within a background of geographic population structure across the species range in Europe and Iran. Although *F*_ST_ estimates were generally low, there was a clear signal of geographic population structure. Subsumed within this geographic variation, there was also a signal of host‐related differentiation, with a small but significant subset of SNPs showing consistent allele frequency differences across sympatric sites. These results suggest that population structure in *R. cerasi* reflects both phylogeographic history and host‐related adaptation, and that host‐association may be restricting gene flow among local *Prunus* and *Lonicera* populations, a mechanism thought to contribute to ecological speciation. Our results demonstrate: (a) that *R. cerasi* populations appear to be genetically diverging based on their use of either *Lonicera* or *Prunus* host plants, and (b) that ddRADseq has increased resolution compared to microsatellites previously used in this system for inferring signatures of host‐associated genetic differentiation.

### 4.1 Geographic population structure

*Rhagoletis cerasi* flies sampled in Europe and Iran genetically grouped into five main geographic clusters, which exhibited an increase in genetic isolation with geographic distance. Several factors most likely contributed to the formation of this pattern. Adult *R. cerasi* flies have short dispersal distances (Boller & Remund, 1983) and produce only one generation per year (Fimiani, 1989). Thus, patchy distributions of host plants and geomorphic barriers such as mountain ranges are expected to limit gene flow. There is also evidence for local adaptation of phenotypes in different geographic populations of *R. cerasi* that may affect genetic differentiation. For example, seasonal timing and metabolic rates of diapausing pupae differ markedly between highland and lowland populations of *R. cerasi* in Greece that are separated by much shorter distances than between many of the populations surveyed in the current study (Papanastasiou et al., 2011).

In both Augustinos et al. (2014) and our study, the most geographically isolated sampling sites found at the edges of the species range were the most genetically diverged and had the lowest nucleotide diversity levels. Similar geographic patterns of genetic differentiation across Europe have been reported in organisms thought to be more dispersive than *R. cerasi*, such as the grey long‐eared bat, *Plecotus austriacus,* where distinct genetic clusters were found in Italy and on the Iberian Peninsula, and the common lizard, *Zootoca vivipara*, where the Pyrenees separate mitochondrial lineages (Milá, Surget‐Groba, Heulin, Gosá, & Fitze, 2013; Razgour et al., 2014). In a contemporary context not accounting for the historic dispersal of this species, it is possible that the central German and Austrian fly populations in our study have higher nucleotide diversity because they reflect a somewhat homogeneous cluster that is susceptible to gene flow from all directions, as opposed to the more remotely isolated sites such as Portugal, Italy, Norway and Iran. An alternative to gene flow is that lineage sorting of shared ancestral polymorphisms has proceeded to differing degrees at different geographic sites related to variation in effective population sizes and the chronology of population divergence (Lawson, Van Dorp, & Falush, 2018).

However, the genetic structure resolved in the current study for *R. cerasi* generally differs from the hypothesised pattern of past dispersal of the fly. In Europe, many species demonstrate geographic clustering of populations around known southern glacial refugia existing during the last glacial maximum, with genetic diversity generally declining in the North with increasing distance from refugia (e.g., Cheddadi et al., 2006). In comparison, we found the greatest genetic diversity for *R. cerasi* in central Europe, with declines in diversity in all geographically radiating directions. This pattern is inconsistent with lingering signatures of postglacial range expansion (Hewitt, 1996), as well as with the migration of *R. cerasi* from the Caucasus area of western Asia (Fimiani, 1989). There are exceptions to this general pattern, however, where different refugial populations interbreed during post glacial expansions, giving rise to higher genetic diversity (Hewitt, 2000). It is also possible that postglacial hybridization contributed to the higher genetic diversity of *R. cerasi* in central Europe. However, the pattern of greater genetic variation for *R. cerasi* in central Europe is more consistent with typical core‐edge dynamics, wherein populations located near southern, western, eastern and northern distribution edges may be more isolated and/or constrained to smaller effective population sizes due to suboptimal environmental conditions compared to the core (reviewed in Hardie & Hutchings, 2010). More intensive sampling in the southern and western portions of the species range will be needed to distinguish among these hypotheses.

### 4.2 Host races of *Rhagoletis cerasi*

Our results support the existence of host‐associated races of *R. cerasi* infesting *Prunus* and *Lonicera*. In the case of relatively high levels of gene flow, we would expect that host‐associated populations would generally genetically cluster based on geography, with a subset of the genome displaying host‐related differentiation (Doellman et al., 2018). We observed this pattern with respect to Norway, but populations within Germany clustered by host plant, not geography. This pattern contrasts with that displayed by *R. pomonella*, where although there is clear evidence for host‐associated differentiation between co‐occurring apple‐ and hawthorn‐infesting populations of the fly across the eastern United States, genetic clustering is largely based on geography and not host plant at similar spatial scales to our sites in Germany (Doellman et al., 2018). It is therefore possible that gene flow is more restricted locally between *Prunus‐* and *Lonicera*‐associated flies in Germany than between sympatric apple and hawthorn populations of *R. pomonella* in the United States. This may reflect the recent host shift of *R. pomonella* to apples with their introduction into the USA, which occurred approximately 170 years ago (Walsh, 1867), whereas there is no evidence for the host shift in *R. cerasi* being as recent. It is also possible that geneflow is more restricted between the host races of *R. cerasi* than in *R. pomonella*, but resolving among these hypotheses will require more exhaustive sequencing of the genome and larger sample sizes coupled with additional approaches, such as mark‐release‐recapture studies (e.g., Feder et al., 1994). Nevertheless, similar cases of local ecological divergence embedded within broader patterns of geographic variation have been reported in several other species including *Timema* stick insects (Soria‐Carrasco et al., 2014), *Gasterosteus* stickleback fish (Colosimo et al., 2005), *Geospiza* Darwin's finches (Hendry, Huber, Leon, Herrel, & Podos, 2009), plant fungal diseases (Giraud, Gladieux, & Gavrilets, 2010), and *Littorina* intertidal snails (Galindo, Morán, & Rolán‐Alvarez, 2009).

A small but significant portion of SNPs displaying host‐associated differentiation between *Prunus‐* and *Lonicera*‐infesting *R. cerasi* flies tended to be consistent across sites, supporting the ecological divergence hypothesis (Nosil, 2012). Consistent patterns of host‐associated divergence across the landscape could be explained by either: (a) repeated and parallel evolution involving multiple host shifts occurring independently at sites involving shared ancestral standing variation (e.g., Doellman et al., 2019; Feder, Berlocher, et al., 2003), or (b) a single, ancestral host shift followed by geographic dispersal across sites (e.g., Cook et al., 2002). We cannot currently resolve between these two hypotheses. The findings that (1) most SNPs displaying significant host‐related allele frequency differences were unique to specific sympatric sites, and (2) *bayenv2* analysis identified only a single host‐associated outlier SNP in common across the German and Norwegian sympatric sites would seem to tentatively favour hypothesis 1 of multiple independent shifts. Such a scenario would explain the uniqueness of most host‐related divergence at sites as reflecting new mutation or local vagaries in the polygenic targets of selection (Doellman et al., 2019; Langerhans & DeWitt, 2004; Ravinet, Prodöhl, & Harrod, 2013). Moreover, if a single host plant shift occurred in *R. cerasi* with subsequent spread, then we might anticipate higher levels of shared differences and a more consistent monophyletic signal than we observed for the fly (Cook et al., 2002). Similar patterns have been found in *Timema* stick insects (Soria‐Carrasco et al., 2014) and in the repeated occurrence of ‘wave’ and ‘crab’ ecotypes of *Littorina* snails (Ravinet et al., 2016). Nevertheless, additional experiments will be required to rigorously distinguish between single and multiple host shifts for *R. cerasi*.

Our current findings differ from those of Schuler et al. (2016), who found no evidence for geographic or host‐related differentiation between *Prunus* and *Lonicera* flies, including the same sympatric samples in Ober‐Ramstadt (DE), based on 13 microsatellite loci. That the highly polymorphic microsatellite loci failed to detect signatures of genetic divergence suggests: (a) that genomic divergence across geography and hosts may be limited to localized regions of the genome not surveyed by the microsatellites, and (b) that gene flow is most likely ongoing and sufficient to homogenise regions of the genome not under geographic selection or divergent selection between sympatric *Prunus* and *Lonicera* flies. Schwarz et al. (2003), however, reported *R. cerasi* host race differences for a single allozyme locus that may be under direct or linked selection, as reported for a number of allozyme loci in *R. pomonella* (Feder, Chilcote, & Bush, 1990b; Feder et al., 1993).

The specific selective factors causing genomic differentiation between *Prunus* and *Lonicera* flies remain unknown. In *R. pomonella*, eclosion time differences that adapt flies to latitudinal and local differences in the fruiting times of apples and hawthorns strongly predict patterns of geographic and host‐related genomic differentiation (Doellman et al., 2018; Egan et al., 2015; Ragland et al., 2015). In contrast to *R. pomonella*, only a slight difference in mean eclosion time exists between *Prunus‐* and *Lonicera*‐infesting flies (Bakovic et al., 2018; Boller & Bush, 1974). However, the pattern of adult eclosion does differ between the two races, with *Prunus* flies eclosing over a shorter period of time than *Lonicera* flies, which emerge slightly later and more gradually (Bakovic et al., 2018; Boller & Bush, 1974). Host fidelity also potentially contributes to non‐random mating and genetic differentiation in *R. cerasi*, with prior adult experience of females to fruit significantly influencing oviposition choice (Boller et al., 1998). Importantly, prior adult experience experiments still resulted in some flies ovipositing into their non‐native host plants (higher preference for *Prunus*). Such patterns of imperfect host fidelity suggest the potential of ongoing gene flow in the field. Finally, it is also possible that endosymbionts could restrict gene flow between the *Prunus* and *Lonicera* host races. If the endosymbiont, *Wolbachia,* infects *Prunus* flies at higher levels than it does *Lonicera* flies, which appears to be the case (Schuler et al., 2016), it will establish a form of unidirectional incompatibility between infected *Prunus* males and uninfected *Lonicera* females, strengthening RI between the host races (Telschow, Flor, Kobayashi, Hammerstein, & Werren, 2007). As such, unidirectional CI can also help account for the pattern of geographic and host‐associated genomic differentiation in *R. cerasi*, as based on our current knowledge of the system, unidirectional CI is expected to reduce gene flow but not stop it entirely (Boller & Bush, 1974). The myriad of potential factors contributing to RI in *R. cerasi* underscores the need for future studies quantifying their individual and collective effects in facilitating the approach of the fly to a tipping point where speciation may be possible (Nosil, Feder, Flaxman, & Gompert, 2017).

### 4.3 Host plant‐associated loci

In this study, we identified 17 SNPs that displayed significant host‐associated divergence in at least two of the three different analyses we performed, meaning that they were differentiated in several sympatric sites. BLAST search queries of these 17 SNP‐containing RAD contigs against the NCBI data base (Nucleotide collection [nr/nt]) largely returned mRNAs for *R. zephyria*, the closest relative to *R. cerasi* whose sequences are currently in the data base. Although we provide a limited number of BLAST hits with no enriched pathways, these SNP queries minimise the possibility that these reads represent contaminants not encoded in the nuclear genome of *R. cerasi*. Future study is needed to determine if these candidate SNPs or surrounding regions contain genes of potential relevancy for host‐associated divergence. However, as has been observed in several other species, outlier SNPs putatively associated with local ecological adaptation were found to be in significantly higher LD with each other in *R. cerasi* than the background LD of all SNPs. For example, Comeault et al. (2016) identified that SNPs associated with colour phenotypes co‐localise on the same linkage group in stick insects. In *R. pomonella*, loci affecting adult eclosion time have been found in putative chromosomal inversions, where reduced recombination among genes can strengthen the effects of divergent selection and act to restrict gene flow below levels for free‐recombining regions of the genome (Feder, Roethele, et al., 2003; Nosil, 2012; Ragland et al., 2017). Higher genetic divergence within inversions has been reported in many species' comparisons (e.g., Basset, Yannic, Brünner, & Hausser, 2006; Besansky et al., 2003) and its implications in the maintenance of species differences has been highlighted (Lohse, Clarke, Ritchie, & Etges, 2015; McGaugh & Noor, 2012; Noor & Bennett, 2009). High LD can also arise between populations as consequences of strong selection and interhost migration, that leads to the buildup of LD under certain migration/selection ratios during particular phases of divergence (Feder & Nosil, 2009; Schilling et al., 2018). In *R. cerasi*, we observe high LD between a subset of outlier SNPs not only between *Prunus* and *Lonicera* populations but within the races as well, similar to what is seen in *Rhagoletis pomonella* (Ragland et al., 2017). This pattern could be caused by divergent selection acting on linked genes or by reduced recombination during meiosis associated with structural variants, such as chromosomal inversions (Stevison, Hoehn, & Noor, 2011). Further study involving genetic crosses and cytology is needed to address the question of the roles of genome structure in affecting host‐related genetic divergence in *R. cerasi*.

In conclusion, we present genetic evidence supporting the existence of host races of *Prunus‐* and *Lonicera*‐infesting *R. cerasi* flies, similar to the host races of *R. pomonella*. Furthermore, our results indicate that both shared standing genetic variation and new mutations specific to local sympatric sites may have contributed to this host shift. Many questions remain and much further work needs to be done on the system to fully understand the nature and interaction of factors generating the patterns of geographic and host‐related genomic differentiation observed in *R. cerasi* and their relationships to RI and speciation. Our results add to a growing list of potential examples of ongoing ecological divergence.

## AUTHOR CONTRIBUTIONS

C.S. and H.S. designed the study, and V.B. H.S. conducted the library preparation. V.B. generated and analysed the data with guidance from G.J.R. and J.L.F., V.B. led the writing of the manuscript. All authors contributed to interpretation of results and critically revising the manuscript.

## Data Availability

Rscripts and appropriate input files (vcf, structure) have been uploaded to Dryad (https://doi.org/10.5061/dryad.8c1g8jf). Complete *Stacks* catalog (consensus sequences) has been uploaded to Dryad (https://doi.org/10.5061/dryad.8c1g8jf). Alignment BAM file generated by mapping ddRADSeq data to *Stacks* catalog has been uploaded to Dryad (https://doi.org/10.5061/dryad.8c1g8jf). Raw DNA sequences have been uploaded to NCBI Genbank SRA (SRR10053858) under the BioProject PRJNA561951.
